# Supplementary material for: Structures of the wild-type MexAB–OprM tripartite pump reveal its complex formation and drug efflux mechanism
Source: Nat Commun. 2019 Apr 3;10:1520. doi: 10.1038/s41467-019-09463-9 (PMC6447562; doi:10.1038/s41467-019-09463-9)
Supplement: Supplementary file 1 — Supplementary Information [file 41467_2019_9463_MOESM1_ESM.pdf]

Supplementary Materials for

Structures of the wild-type MexAB-OprM tripartite pump reveal its complex formation and drug efflux mechanism.

Tsutsumi *et al.*

Supplementary Figure 1

(a)

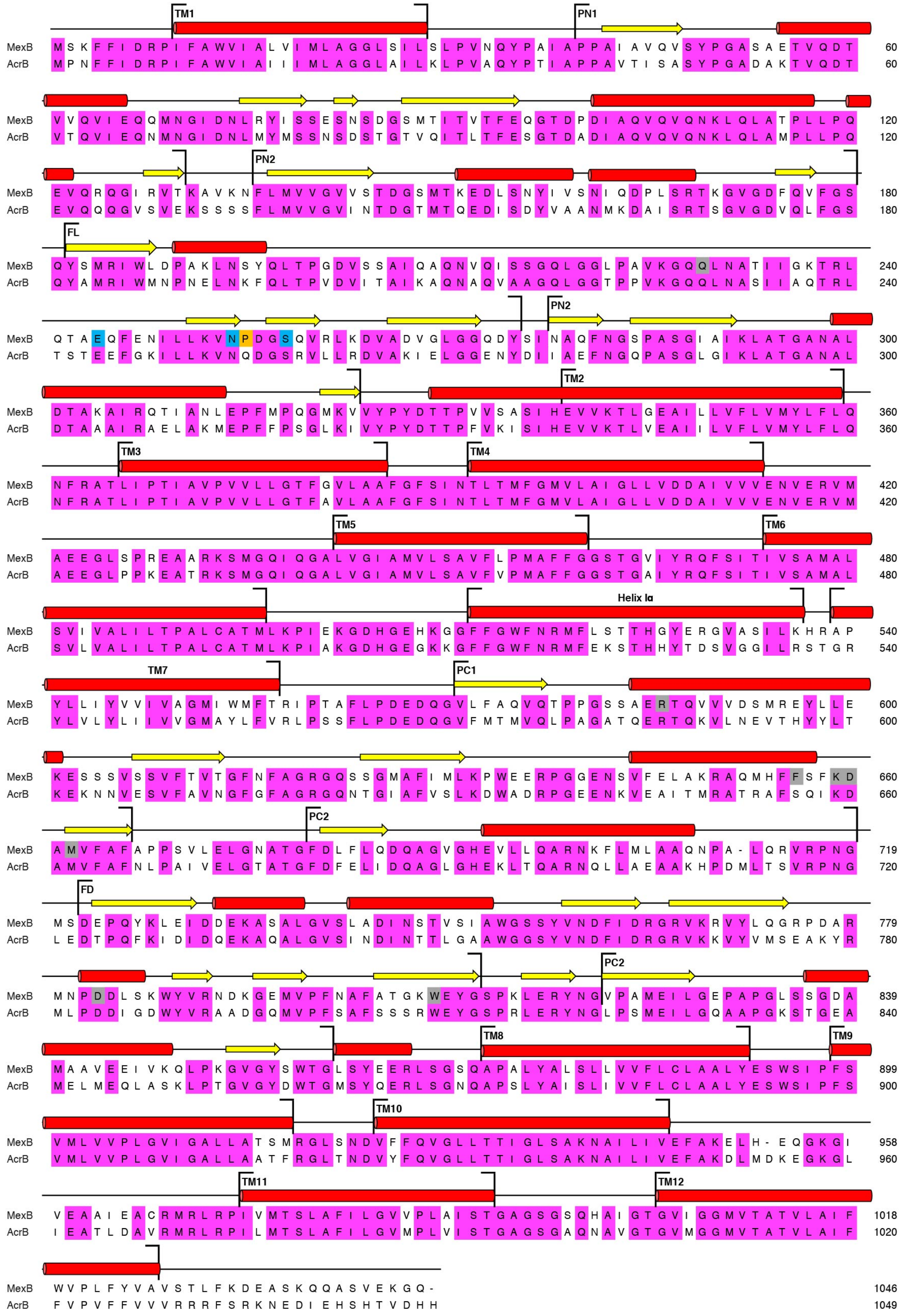

(b)

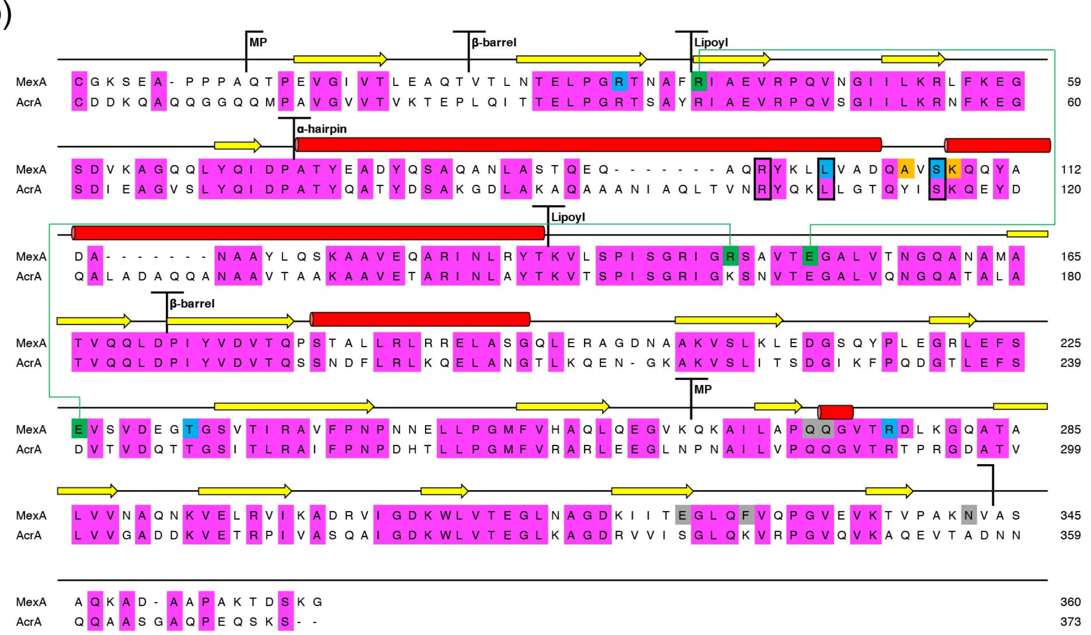
MP
β-barrel
Lipoyl
MexA CGKSEA-PPPAQTPEVGIVTLEAQTVTLNTELPGRTNAFRIAEVRPQVNGIILKRLFKEG 59
AcrA CDDKQAQQGGQQMPAVGVVTVKTEPLQITTELPGRTSAYRIAEVRPQVSGIILKRNFKEG 60
α-hairpin
MexA SDVKAGQQLYQIDPATYEADYQSAQANLASTQEQ-------AQRYKLLVADQAVSKQQYA 112
AcrA SDIEAGVSLYQIDPATYQATYDSAKGDLAKAQAAANIAQLTVNRYQKLLGTQYISKQEYD 120
Lipoyl
MexA DA-------NAAYLQSKAAVEQARINLRYTKVLSPISGRIGRSAVTEGALVTNGQANAMA 165
AcrA QALADAQQANAAVTAAKAAVETARINLAYTKVTSPISGRIGKSNVTEGALVQNGQATALA 180
β-barrel
MexA TVQQLDPIYVDVTQPSTALLRLRRELASGQLERAGDNAAKVSLKLEDGSQYPLEGRLEFS 225
AcrA TVQQLDPIYVDVTQSSNDFLRLKQELANGTLKQEN-GKAKVSLITSDGIKFPQDGTLEFS 239
MP
MexA EVSVDEGTGSVTIRAVFPNPNNELLPGMFVHAQLQEGVKQKAILAPQQGVTRDLKGQATA 285
AcrA DVTVDQTTGSITLRAIFPNPDHTLLPGMFVRARLEEGLNPNAILVPQQGVTRTPRGDATV 299
MexA LVVNAQNKVELRVIKADRVIGDKWLVTEGLNAGDKIITEGLQFVQPGVEVKTVPAKNVAS 345
AcrA LVVGADDKVETRPIVASQAIGDKWLVTEGLKAGDRVVISGLQKVRPGVQVKAQEVTADNN 359
MexA AQKAD-AAPAKTDSKG 360
AcrA QQAASGAQPEQSKS-- 373

(c)

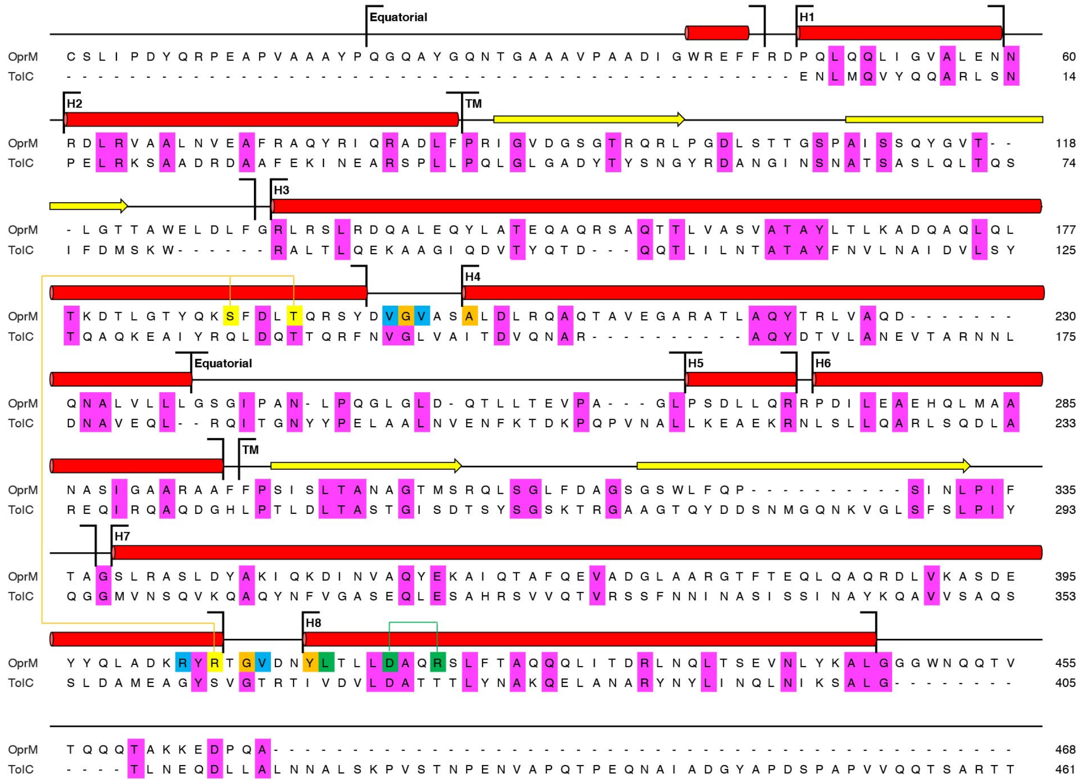
Equatorial
H1
OprM CSLIPDYQRPEAPVAAAYPQGQAYGQNTGAAAVPAADIGWREFFRDPQLQQLIGVALENN 60
TolC --ENLMQVYQQARLSN 14
H2
TM
OprM RDLRVAALNVEAFRAQYRIQRADLFPRIGVDGSGTRQRLPGDLSTTGSPAISSQYGVT-- 118
TolC PELRKSAADRDAAFEKINEARSPLLPQLGLGADYTYSNGYRDANGINSNATSASLQLTQS 74
H3
OprM -LGTTAWELDLFGRLRSLRDQALEQYLATEQAQRSAQTTLVASVATAYLTLKADQAQLQL 177
TolC IFDMSKW------RALTLQEKAAGIQDVTYQTD---QQTLILNTATAYFNVLNAIDVLSY 125
H4
OprM TKDTLGTYQKSFDLTQRSYDVGVASALDLRQAQTAVEGARATLAQYTRLVAQD------- 230
TolC TQAQKEAIYRQLDQTTQRFNVGLVAITDVQNAR----------AQYDTVLANEVTARNNL 175
Equatorial
H5
H6
OprM QNALVLLLGSGIPAN-LPQGLGLD-QTLLTEVPA---GLPSDLLQRRPDILEAEHQLMAA 285
TolC DNAVEQL--RQITGNYYPELAALNVENFKTDKPQPVNALLKEAEKRNLSLLQARLSQDLA 233
TM
OprM NASIGAARAAFFPSISLTANAGTMSRQLSGLFDAGSGSWLFQP----------SINLPIF 335
TolC REQIRQAQDGHLPTLDLTASTGISDTSYSGSKTRGAAGTQYDDSNMGQNKVGLSFSLPIY 293
H7
OprM TAGSLRASLDYAKIQKDINVAQYEKAIQTAFQEVADGLAARGTFTEQLQAQRDLVKASDE 395
TolC QGGMVNSQVKQAQYNFVGASEQLESAHRSVVQTVRSSFNNINASISSINAYKQAVVSAQS 353
H8
OprM YYQLADKRYRTGVDNYLTLLDAQRSLFTAQQQLITDRLNQLTSEVNLYKALGGGWNQQTV 455
TolC SLDAMEAGYSVGTRTIVDVLDATTTLYNAKQELANARYNYLINQLNIKSALG-------- 405
OprM TQQQTAKKEDPQA--- 468
TolC ----TLNEQDLLALNNALSKPVSTNPENVAPQTPEQNAIADGYAPDSPAPVVQQTSARTT 461

Supplementary Figure 1. Sequence alignment of multidrug efflux pumps.

Sequence alignment of RND (a), MFP (b), and OMF (c) by Clustal Omega[1]. Conserved residues are marked with magenta. Residues whose side chains are interact with other components are shown in blue. Residues whose main chains interact with other components are shown magenta. Residues marked with green and connecters represent interactions between the corresponding proteins. Yellow residues with connecters represent interactions within a protein. Gray markers represent residues that form the LC protomer–MexB interaction surface.

Supplementary Figure 2

(a)

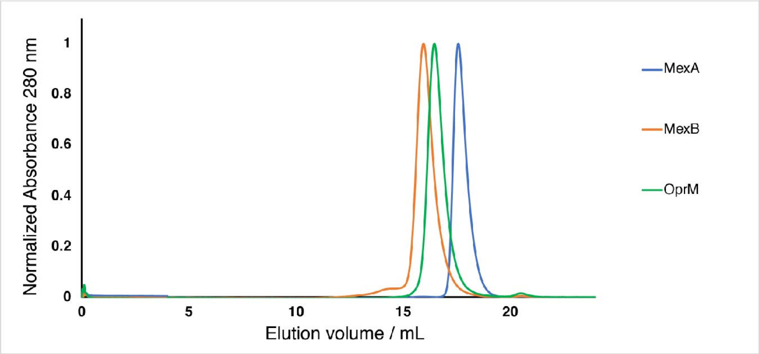

(b)

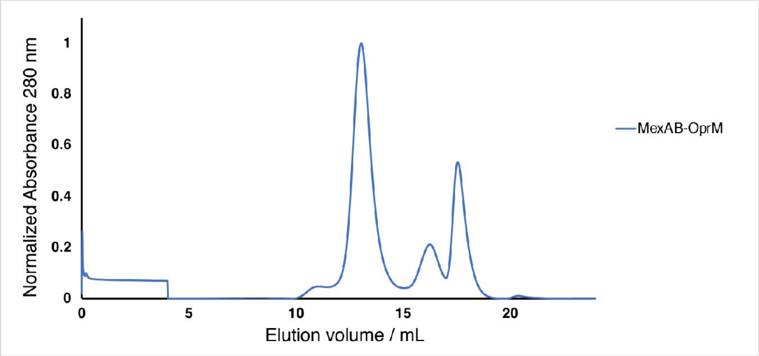

(c)

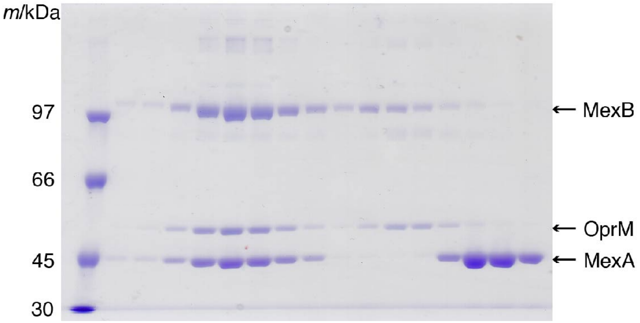

Supplementary Figure 2. Reconstruction of MexAB–OprM.

(a) SEC chart of MexA, MexB, and OprM on a Superose6 Increase 10/300 column with buffer C (see Methods). The flow-rate is 0.5 mL min^{-1}. (b) SEC chart of the reconstructed

sample on Superose6 Increase 10/300 column with 20 mM Na-citrate, 300 mM KCl, 0.02% CYMAL-7. (c) SDS-PAGE of (b) fractions.

Supplementary Figure 3

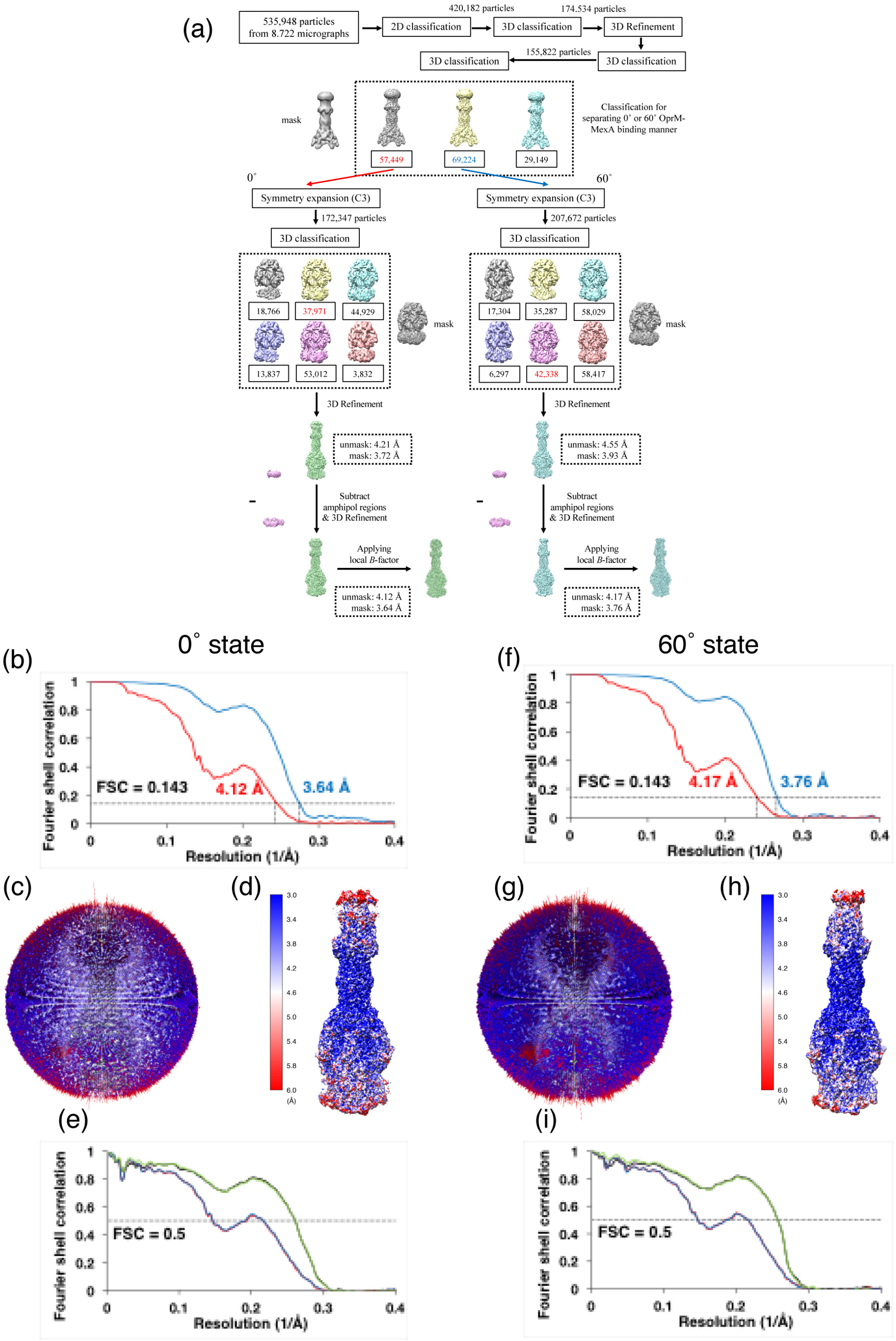

Supplementary Figure 3. Single-particle analysis of the apo-state.

(a) Overview of image processing, 3D reconstruction, and map sharpening (see Methods). (b)–(i) Maps and model validation of the 0˚ state (b–e) and 60˚ state (f–i) of apo-state MexAB–OprM. (b, f) Fourier shell correlation (FSC) curves before (red) and after (blue) postprocessing. (c, g) Angular distribution histograms. (d, h) Local resolution estimation, calculated using ResMap. (e, i) FSC curves for final model versus final locscale map (black), masked map (green), half map 1 (red), and half map 2 (blue), respectively.

Supplementary Figure 4

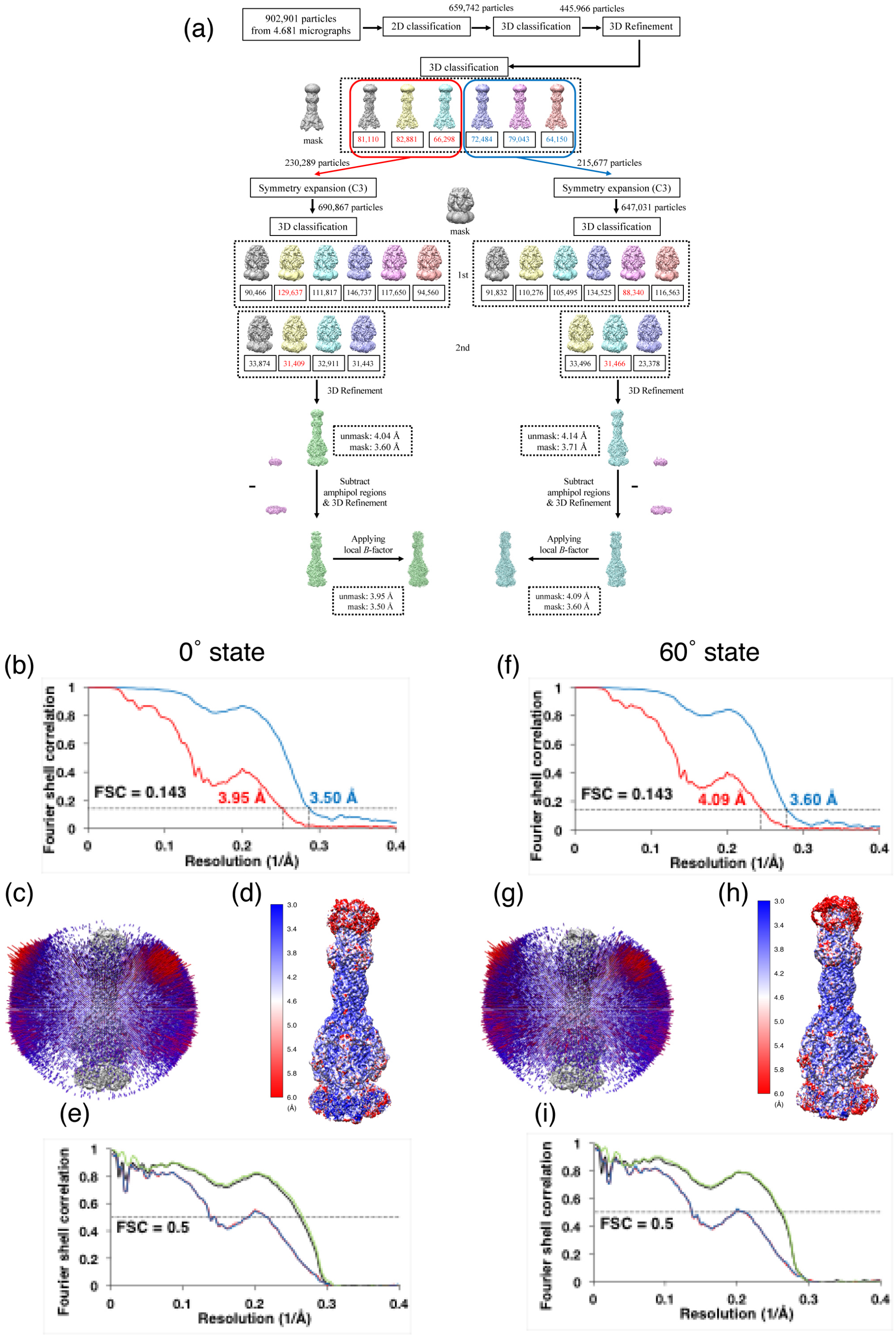

Supplementary Figure 4. Single-particle analysis of the novobiocin-binding state.

(a) Overview of image processing, 3D reconstruction, and map sharpening (see Methods). (b–i) Maps and model validation of the 0˚ state (b–e) and 60˚ state (f–i) of the novobiocin-binding state of MexAB-OprM. (b, f) FSC curves before (red) and after (blue) postprocessing. (c, g) Angular distribution histograms. (d, h) Local resolution estimation calculated using ResMap. (e, i) FSC curves for final model versus final locscale map (black), masked map (green), half map 1 (red), and half map 2 (blue), respectively.

Supplementary Figure 5

(a)

Interaction	Protein	Mutation	Result
MexA-MexA interaction	MexA	R39A	○
		R39D	×
		R147A	○
		R147D	×
		Q168A	○
		E211A	○
MexA-OprM interaction	MexA	R96A	○
		R96D	△*1
		L99D	○
		L100D	×
		D103A	○
		Q104A	○
		S107D	×
		K108A	○
	OprM	R194A	○
		V198D	○
		G199A	×
		V200D	○
		R403A	×
		G407A	×
		V408D	○
		N410A	○
MexA-MexB interaction	MexA	R34A	×
		R34D	×
		T233A	×
		T233V	×
		R277A	×
		R277D	×

*1: The efficiency for complex formation was decreased.

(b)

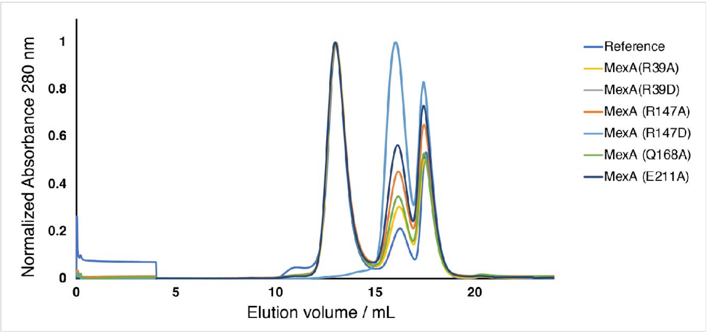

(c)

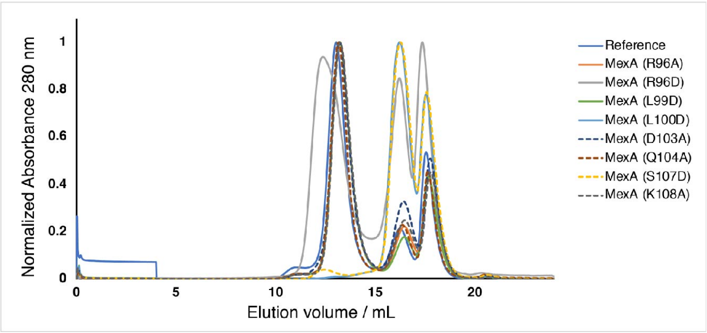

(d)

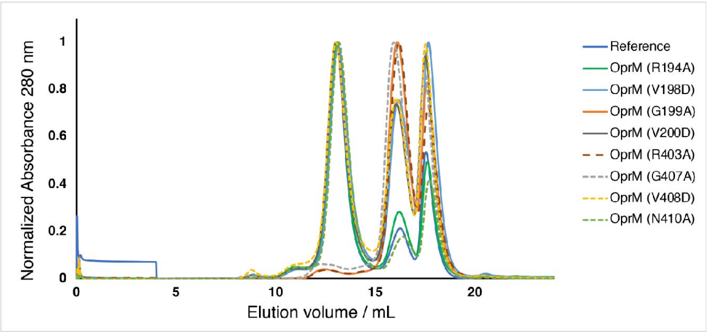

(e)

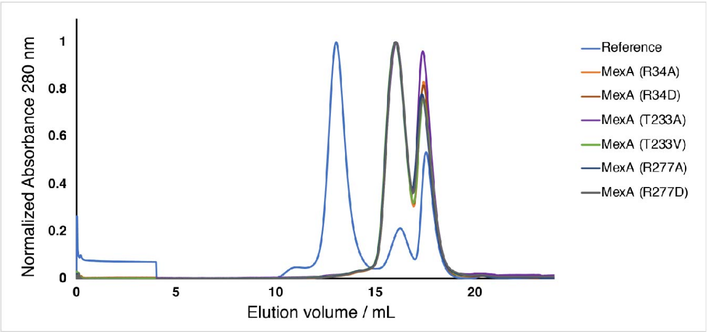

Supplementary Figure 5. Complex formation experiment with point mutation and SEC

(a) Summary of the complex formation experiment. Leftmost panels show the interaction surfaces to which each residue is related. Middle left panels represent mutated proteins. Middle right panels show the type of mutation added to each residue. Rightmost panels show the results of complex formation. A circle indicates that the complex formed in the same way as the wild-type. A cross indicates that no complex was formed. A white triangle indicates that the efficiency of complex formation was reduced. A black triangle indicates that the stability of the reconstructed MexAB–OprM complex was reduced. (b)–(e) SEC chart of residues in MexA involved in the MexA–MexA interaction (b), the MexA–OprM interaction (c), residues in OprM (d), and residues in MexA involved in the MexA–MexB interaction (e). The conditions of SEC are as described in Fig. S2.

Supplementary Figure 6

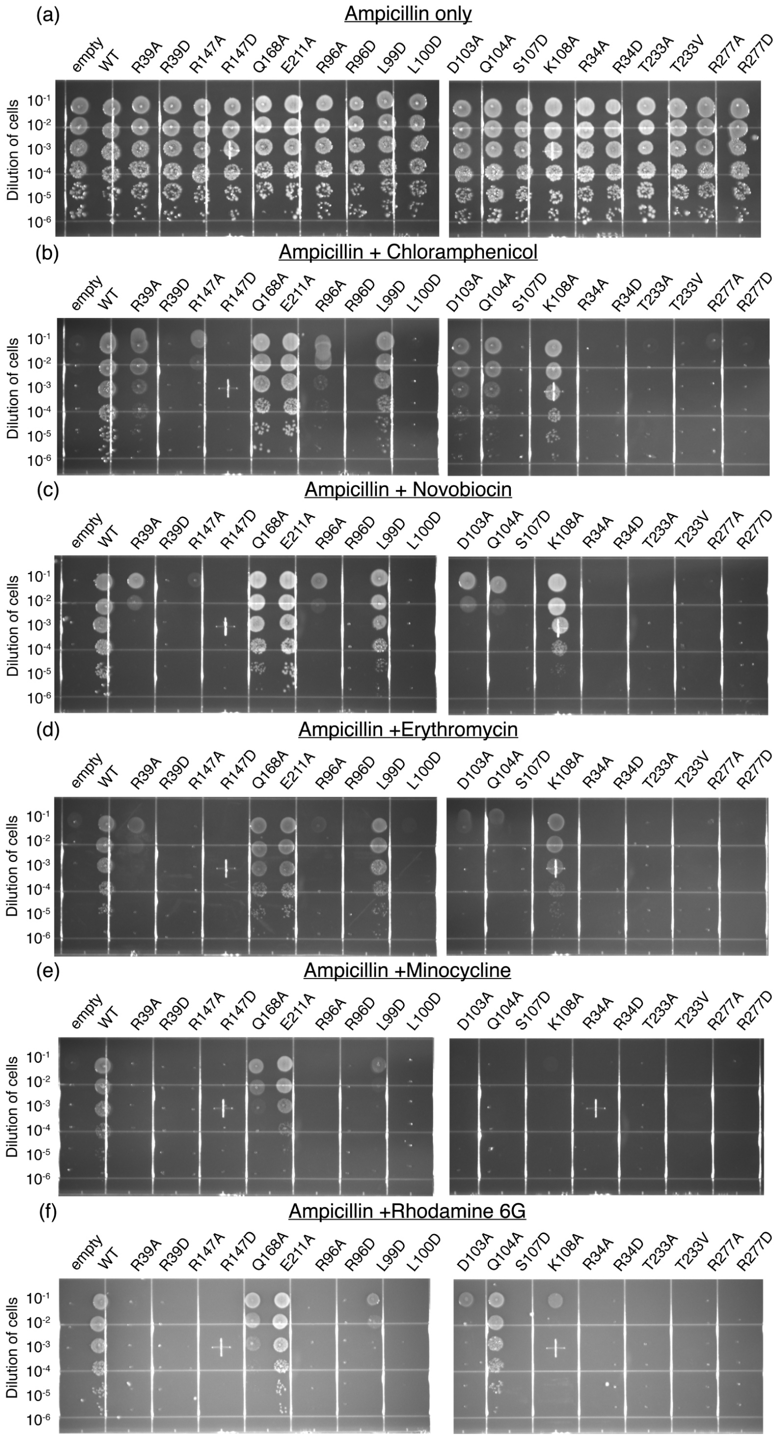

Supplementary Figure 6. The drug resistance assay using MexA mutants.

Representative images of serial dilutions of cells plated on LB agar supplemented with 100 μg mL^{-1} of ampicillin and an additional antibiotic: none (a), 5 μg mL^{-1} of chloramphenicol (b), 25 μg mL^{-1} of novobiocin (c), 5 μg mL^{-1} of erythromycin (d), 0.5 μg mL^{-1} of minocycline (e), or 10 μg mL^{-1} of rhodamine 6G (f). The top panels indicate residues in MexA which are mutated.

Supplementary Figure 7

(a)

(b)

(c)

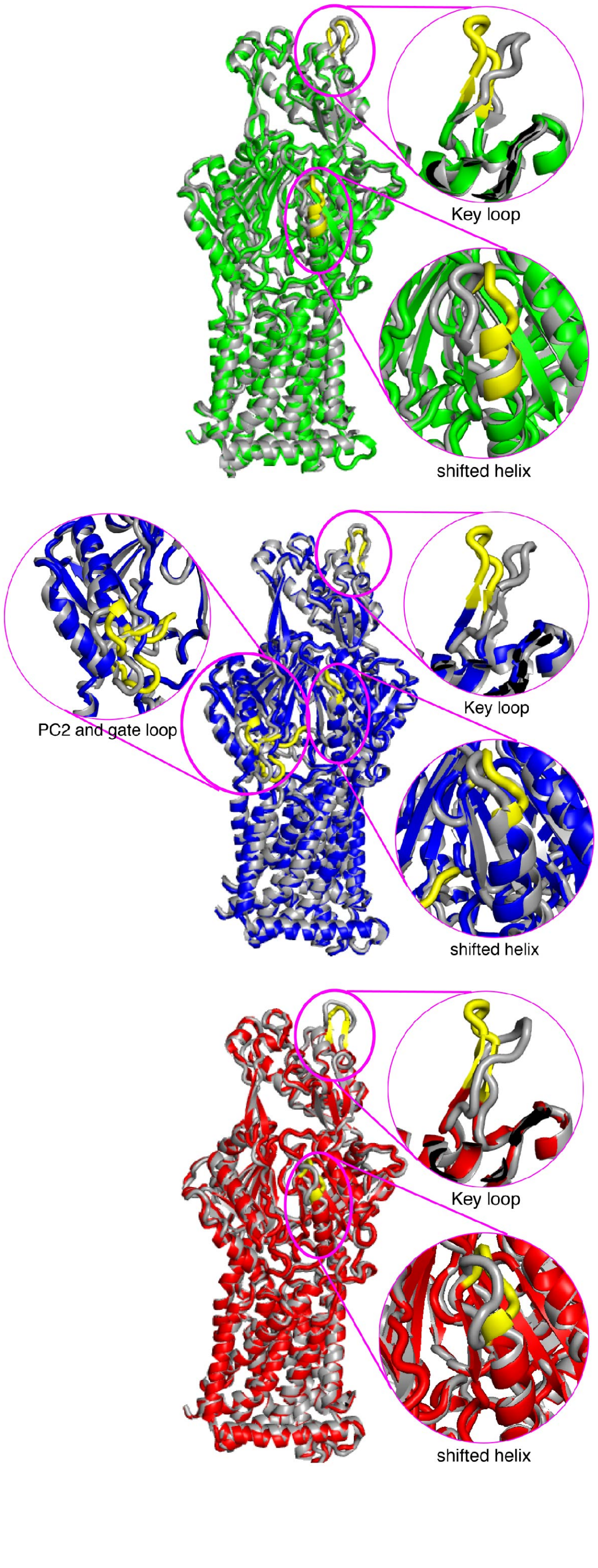

Supplementary Figure 7. Comparison of individual MexB protomers in the apo-MexAB–OprM or isolated crystal structure.

Superposition of each MexB protomer in the crystal structure (PDBID: 3w9i) and complex structure (apo-state, 0˚). (a) Superposition of Access protomers in the apo-MexAB-OprM (green) and the crystal structure (gray). (b) Superposition of the resting protomer in apo-MexAB–OprM (blue) and the Binding protomer in the crystal structure (gray). (c) Superposition of Extrusion protomers in apo-MexAB–OprM (red) and the crystal structure (gray).

Supplementary Figure 8

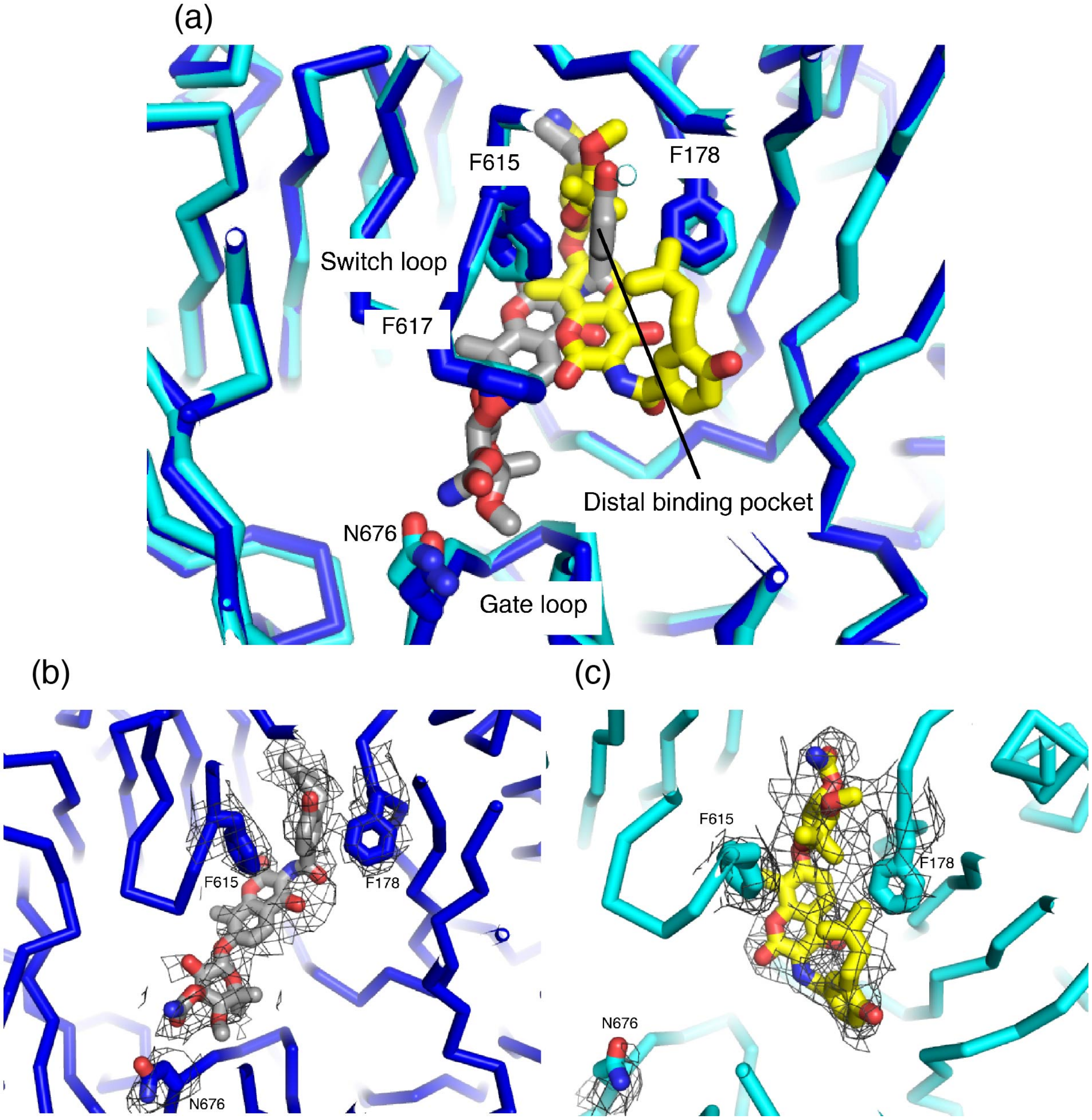

Supplementary Figure 8. Binding modes in the distal binding pocket of the novobiocin-binding state.

(a) Superposed view of the distal binding pocket of the MexB protomer in the Binding state in the 0˚ state (blue) and the 60˚ state (cyan). Models of novobiocin are shown in gray

(0˚ state) and yellow (60˚ state). (b) and (c) Map and model fit around novobiocin in the 0˚ state (b) and the 60˚ state (c). Cryo-EM map within 1.6 Å around novobiocin, shown as a gray mesh contoured at 3.0 σ. Models are colored as (a).

Supplementary Figure 9

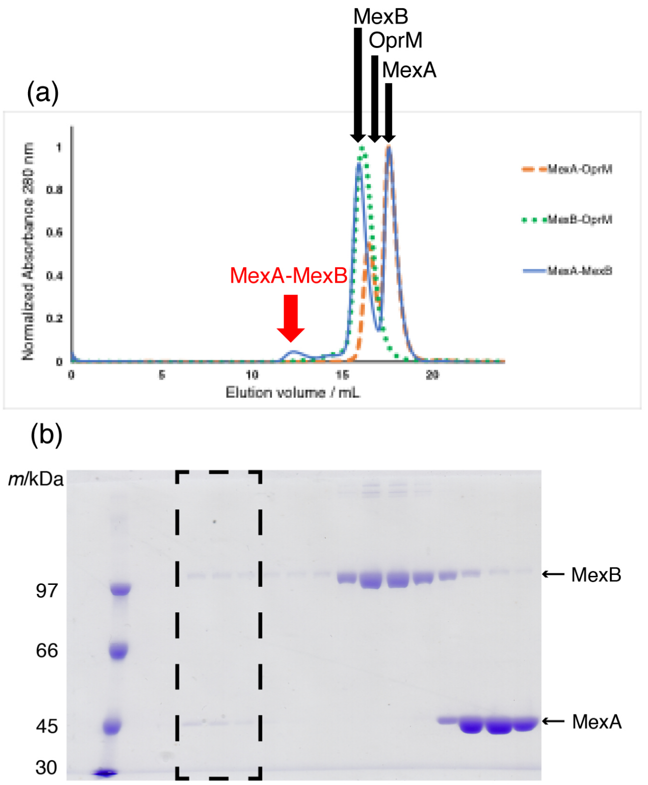

Supplementary Figure 9. Reconstruction of the MexA–MexB complex.

(a) SEC charts of samples dialyzed in the same manner as in the complex formation experiment, except that only MexA and MexB (blue), MexA and OprM (orange), or MexB and OprM (green) were present. Red arrow indicates a peak corresponding to the MexA–MexB complex. (b) SDS-PAGE of fractions of the MexA–MexB sample. A black dashed box indicates fractions corresponding to the peak shown in (a).

Supplementary Figure 10

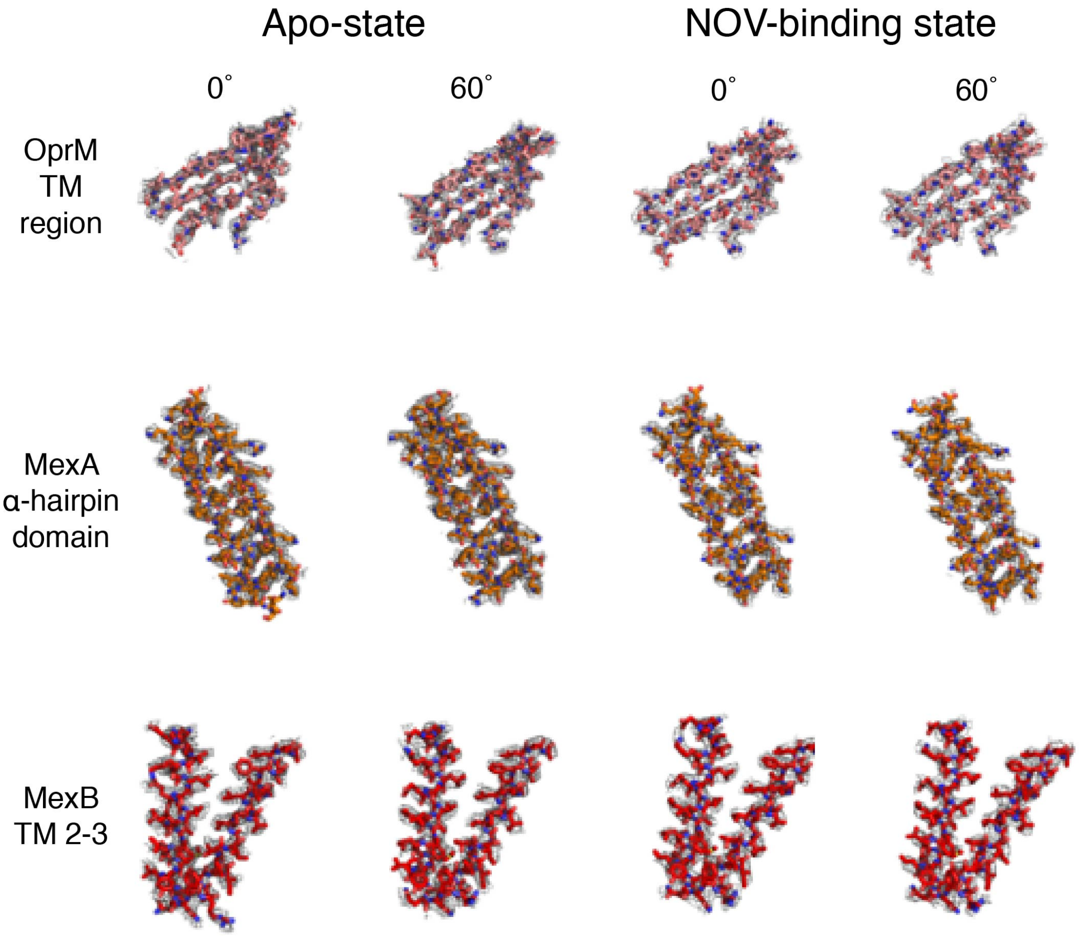

Supplementary Figure 10. Representative cryo-EM maps and fitted models.

Top panel: β-barrel domain in OprM (a.a. 87–96, 114–127, 299–310, and 321–331). Middle panel: α-hairpin domain in MexA (73–136). Bottom panel: transmembrane helixes 2 and 3 in MexB (a.a. 338–385). The two left columns show segments from the apo-state structure, and the two right columns show segments from the novobiocin-binding state

structure. Leftmost and middle right columns represent the 0˚ state structure, and middle left and rightmost columns represent the 60˚ state structure. Cryo-EM maps within 1.6 Å around models are shown as gray meshes contoured at 3.0 σ.

Supplementary Figure 11

(a) 0˚ state

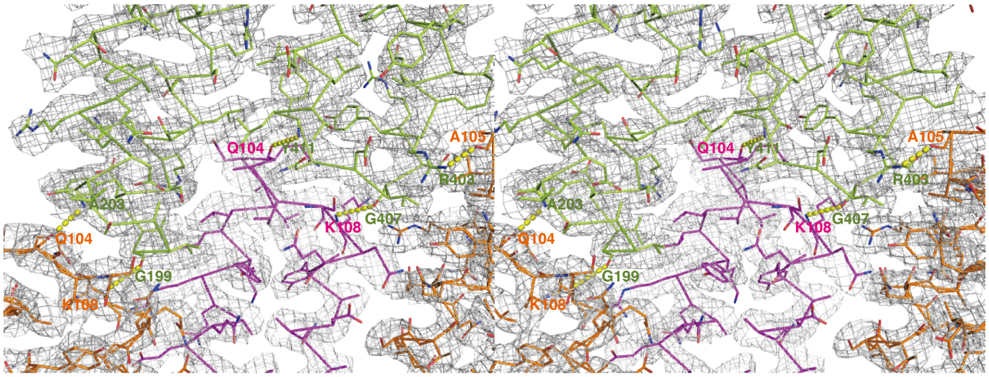

(b) 60˚ state

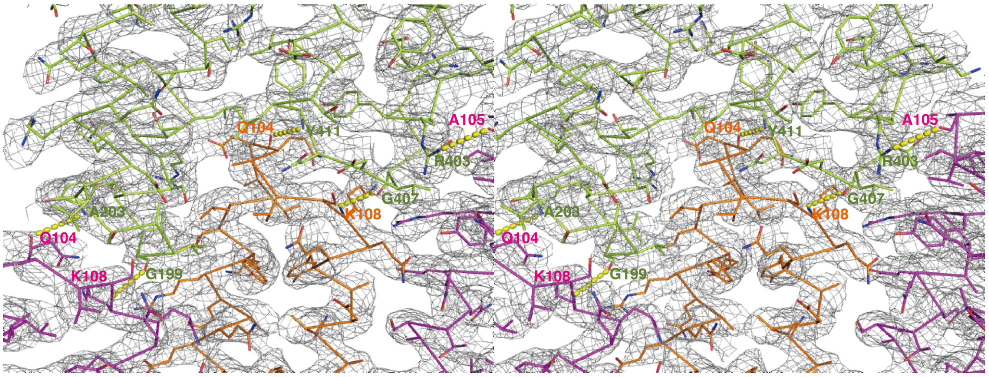

Supplementary Figure 11. Stereo Views of Cryo-EM maps and fitted models of interaction areas between MexA and OprM

Stereo views of cryo-EM maps and fitted models of the α-hairpin of OprM and the α-hairpin of MexA in the 0˚ state (a) or 60˚ state (b). Each protomer is colored as in Fig. 2. Cryo-EM maps are shown as a gray mesh contoured at 3.0 σ.

Supplementary Table 1. Summary of MexAB–OprM structure determined by cryo-EM single-particle analysis.

	Apo state (0˚) EMDB: EMD-9695 PDB: 6IOK	Apo state (60˚) EMDB: EMD-9696 PDB: 6IOL	NOV binding state (0˚)	NOV binding state (60˚)
**Data collection and processing**				
Microscope	FEI Titan Krios			
Magnification	75,000x		59,000x	
Voltage (kV)	300			
Electron exposure (e^{-}/Å^{2})	40		40	
Detector	FEI Falcon Ⅱ		FEI Falcon Ⅲ (linear mode)	
Pixel size (Å/pixel)	0.875		1.125	
Defocus range (μm)	-1.25 to -3.0		-1.25 to -2.5	
Number of used micrographs	8,722		4,681	
Initial number of particles	535,948		902,901	
Final number of particles for reconstruction	37,971	42,338	31,409	31,466
Symmetry imposed	C1	C1	C1	C1
Box size (pixels)	400		320	
Map resolution (Å)	3.64	3.76	3.50	3.60
FSC threshold	0.143	0.143	0.143	0.143
**Refinement**				
Model composition				
Number of non-H atoms	49,014	49,077	49,126	49,126
Protein residues	6,441	6,450	6,453	6,453
Ligands	-	-	1	1
B factors (Å^{2})				
Proteins	24.49	22.99	88.04	96.24
Ligands	-	-	87.92	20.00
R.M.S deviations				
Bond lengths (Å)	0.007	0.005	0.017	0.006
Bond angles (˚)	1.036	0.961	1.130	1.107
Ramachandran plot				
Favored (%)	95.40	94.44	93.62	94.46
Allowed (%)	4.60	5.56	6.38	5.54
Outliers (%)	0	0	0	0
Validiation				
MolProbity score	1.59	1.65	1.71	1.64
Clashscore	5.10	4.68	5.38	5.03
Poor rotamers (%)	0.35	0.33	0.96	0.83
Model resolution (Å)	3.85	3.89	3.83	3.87
FSC threthold	0.5	0.5	0.5	0.5
Real Space CC	0.757	0.760	0.766	0.770

Supplementary Table 2. Lists of primers used in this study.

Primer name	Sequences (5’-3’)
MexA_Forward	AACATATGGCCGAGTCGAGCGGAAAAAG
MexA_Reverse	AACTCGAGTCAGCCCTTGCTGTCGG
OprM_Forward	CGCATATGAAACGGTCCTTCCTTTCC
OprM_Reverse	AAAAGCTTTCAGTGGTGATGGTGGTGATG
MexA_R34A_Forward	GAATACCGAGCTGCCGGGCGCGACCAATGCGTTCCGCATC
MexA_R34A_Reverse	GATGCGGAACGCATTGGTCGCGCCCGGCAGCTCGGTATTC
MexA_R34D_Forward	GAATACCGAGCTGCCGGGCGATACCAATGCGTTCCGCATCG
MexA_R34D_Reverse	CGATGCGGAACGCATTGGTATCGCCCGGCAGCTCGGTATTC
MexA_R39A_Forward	GGCCGGACCAATGCGTTCGCCATCGCCGAGGTGCGTCC
MexA_R39A_Reverse	GGACGCACCTCGGCGATGGCGAACGCATTGGTCCGGCC
MexA_R39D_Forward	GGCCGGACCAATGCGTTCGACATCGCCGAGGTGCGTCC
MexA_R39D_Reverse	GGACGCACCTCGGCGATGTCGAACGCATTGGTCCGGCC
MexA_R96A_Forward	CCCAGGAACAGGCCCAGGCCTACAAGCTGCTGGTCG
MexA_R96A_Reverse	CGACCAGCAGCTTGTAGGCCTGGGCCTGTTCCTGGG
MexA_R96D_Forward	CCCAGGAACAGGCCCAGGATTACAAGCTGCTGGTCGC
MexA_R96D_Reverse	GCGACCAGCAGCTTGTAATCCTGGGCCTGTTCCTGGG
MexA_L99D_Forward	CAGGCCCAGCGCTACAAGGATCTGGTCGCCGACCAGGC
MexA_L99D_Reverse	GCCTGGTCGGCGACCAGATCCTTGTAGCGCTGGGCCTG
MexA_L100D_Forward	CCCAGCGCTACAAGCTGGATGTCGCCGACCAGGCCGTG
MexA_L100D_Reverse	CACGGCCTGGTCGGCGACATCCAGCTTGTAGCGCTGGG
MexA_D103A_Forward	CAAGCTGCTGGTCGCCGCCCAGGCCGTGAGCAAGC
MexA_D103A_Reverse	GCTTGCTCACGGCCTGGGCGGCGACCAGCAGCTTG
MexA_Q104A_Forward	CAAGCTGCTGGTCGCCGACGCGGCCGTGAGCAAGCAGC
MexA_Q104A_Reverse	GCTGCTTGCTCACGGCCGCGTCGGCGACCAGCAGCTTG
MexA_S107D_Forward	GTCGCCGACCAGGCCGTGGACAAGCAGCAGTACGCCG
MexA_S107D_Reverse	CGGCGTACTGCTGCTTGTCCACGGCCTGGTCGGCGAC
MexA_K108A_Forward	CCGACCAGGCCGTGAGCGCGCAGCAGTACGCCGACG

MexA_K108A_Reverse	CGTCGGCGTACTGCTGCGCGCTCACGGCCTGGTCGG
MexA_R147A_Forward	GATCTCCGGCCGCATCGGCGCTTCCGCGGTGACCGAAGG
MexA_R147A_Reverse	CCTTCGGTCACCGCGGAAGCGCCGATGCGGCCGGAGATC
MexA_R147D_Forward	GATCTCCGGCCGCATCGGCGATTCCGCGGTGACCGAAGG
MexA_R147D_Reverse	CCTTCGGTCACCGCGGAATCGCCGATGCGGCCGGAGATC
MexA_Q168A_Forward	CAACGCGATGGCCACCGTGGCACAGCTCGACCCGATCTAC
MexA_Q168A_Reverse	GTAGATCGGGTCGAGCTGTGCCACGGTGGCCATCGCGTTG
MexA_E211A_Forward	GAAGGTCTCCCTGAAGCTGGCGGACGGTAGCCAATACCCG
MexA_E211A_Reverse	CGGGTATTGGCTACCGTCCGCCAGCTTCAGGGAGACCTTC
MexA_T233A_Forward	GTTTCCGTCGACGAAGGCGCCGGCTCGGTCACCATCC
MexA_T233A_Reverse	GGATGGTGACCGAGCCGGCGCCTTCGTCGACGGAAAC
MexA_T233V_Forward	GTTTCCGTCGACGAAGGCGTCGGCTCGGTCACCATCCG
MexA_T233V_Reverse	CGGATGGTGACCGAGCCGACGCCTTCGTCGACGGAAAC
MexA_R277A_Forward	CCGCAGCAAGGCGTGACCGCCGACCTCAAGGGCCAGGC
MexA_R277A_Reverse	GCCTGGCCCTTGAGGTCGGCGGTCACGCCTTGCTGCGG
MexA_R277D_Forward	CCGCAGCAAGGCGTGACCGACGACCTCAAGGGCCAGGC
MexA_R277D_Reverse	GCCTGGCCCTTGAGGTCGTCGGTCACGCCTTGCTGCGG
OprM_R194A_Forward	GAGTTTCGACCTGACCCAGGCCAGCTACGACGTCGGCG
OprM_R194A_Reverse	CGCCGACGTCGTAGCTGGCCTGGGTCAGGTCGAAACTC
OprM_V198D_Forward	CCAGCGCAGCTACGACGACGGCGTCGCCTCCGCGC
OprM_V198D_Reverse	GCGCGGAGGCGACGCCGTCGTCGTAGCTGCGCTGG
OprM_G199A_Forwar	GCGCAGCTACGACGTCGCCGTCGCCTCCGCGCTCG

d	
OprM_G199A_Reverse	CGAGCGCGGAGGCGACGGCGACGTCGTAGCTGCGC
OprM_V200D_Forward	CAGCTACGACGTCGGCGACGCCTCCGCGCTCGACC
OprM_V200D_Reverse	GGTCGAGCGCGGAGGCGTCGCCGACGTCGTAGCTG
OprM_R403A_Forward	CTACCAGCTCGCCGACAAGGCCTATCGCACGGGGGTGG
OprM_R403A_Reverse	CCACCCCCGTGCGATAGGCCTTGTCGGCGAGCTGGTAG
OprM_G407A_Forward	GACAAGCGCTATCGCACGGCGGTGGACAACTACCTGACC
OprM_G407A_Reverse	GGTCAGGTAGTTGTCCACCGCCGTGCGATAGCGCTTGTC
OprM_V408D_Forward	GCGCTATCGCACGGGGGATGACAACTACCTGACCCTG
OprM_V408D_Reverse	CAGGGTCAGGTAGTTGTCATCCCCCGTGCGATAGCGC
OprM_N410A_Forward	CTATCGCACGGGGGTGGACGCCTACCTGACCCTGCTCG
OprM_N410A_Reverse	CGAGCAGGGTCAGGTAGGCGTCCACCCCCGTGCGATAG

Supplementary Table 3. Lists of strains and plasmids used in this study.

Strain or plasmid	Genotype or characteristics	Reference
E. coli		
BL21(DE3)RIPL	F^- *ompT hsdS*(r_B^- m_B^-) *dcm*$^+$ Tet^r *gal* λ(DE3) *endA* Hte [*argU proL* Cam^r] [*argU ileY leuW* Strep/Spec^r]	Stratagene
C43(DE3)	F^- *ompT* $hsdS_B$ (r_B^- m_B^-) *gal dcm* (DE3)	OverExpress
**Plasmids**		
pAzu-MexA	Cloning template for *mexA*	[2]
pOprM-His	Cloning template for *oprM*	[3]
pET21b+	Protein expression vector (Ap^r)	Novagen
pET28b+	Protein expression vector (Km^r)	Novagen
pET-MexA	Plasmid for overexpression of MexA with N-terminal His tag	This study
pET-OprM	Plasmid for overexpression of OprM with C-terminal His tag	This study
pMexB	Plasmid for overexpression of MexB with C-terminal His tag	[4]
pET-MexA-R34A	Plasmid for overexpression of MexA-R34A	This study
pET-MexA-R34D	Plasmid for overexpression of MexA-R34D	This study
pET-MexA-R39A	Plasmid for overexpression of MexA-R39A	This study
pET-MexA-R39D	Plasmid for overexpression of MexA-R39D	This study
pET-MexA-R96A	Plasmid for overexpression of MexA-R96A	This study
pET-MexA-R96D	Plasmid for overexpression of MexA-R96D	This study
pET-MexA-L99D	Plasmid for overexpression of MexA-L99D	This study
pET-MexA-L100D	Plasmid for overexpression of MexA-L100D	This study
pET-MexA-D103A	Plasmid for overexpression of MexA-D103A	This study
pET-MexA-Q104A	Plasmid for overexpression of MexA-Q104A	This study
pET-MexA-S107D	Plasmid for overexpression of MexA-S107D	This study
pET-MexA-K108A	Plasmid for overexpression of MexA-K108A	This study
pET-MexA-R147A	Plasmid for overexpression of MexA-R147A	This study
pET-MexA-R147D	Plasmid for overexpression of MexA-R147D	This study
pET-MexA-Q168A	Plasmid for overexpression of MexA-Q168A	This study
pET-MexA-E211A	Plasmid for overexpression of MexA-E211A	This study
pET-MexA-T233A	Plasmid for overexpression of MexA-T233A	This study
pET-MexA-T233V	Plasmid for overexpression of MexA-T233V	This study
pET-MexA-R277A	Plasmid for overexpression of MexA-R277A	This study
pET-MexA-R277D	Plasmid for overexpression of MexA-R277D	This study
pET-OprM-R194A	Plasmid for overexpression of OprM-R194A	This study
pET-OprM-V198D	Plasmid for overexpression of OprM-V198D	This study
pET-OprM-G199A	Plasmid for overexpression of OprM-G199A	This study
pET-OprM-V200D	Plasmid for overexpression of OprM-V200D	This study

pET-OprM-R403A	Plasmid for overexpression of OprM-R403A	This study
pET-OprM-G407A	Plasmid for overexpression of OprM-G407A	This study
pET-OprM-V408D	Plasmid for overexpression of OprM-V408D	This study
pET-OprM-N410A	Plasmid for overexpression of OprM-N410A	This study

Supplementary References

1. Sievers, F. & Higgins, D. G. Clustal Omega. *Curr. Protoc. Bioinforma.* **2014**, 3.13.1-3.13.16 (2014).

2. Akama, H. *et al.* Crystal structure of the membrane fusion protein, MexA, of the multidrug transporter in Pseudomonas aeruginosa. *J. Biol. Chem.* **279**, 25939–25942 (2004).

3. Akama, H. *et al.* Crystal structure of the drug discharge outer membrane protein, OprM, of Pseudomonas aeruginosa: Dual modes of membrane anchoring and occluded cavity end. *J. Biol. Chem.* **279**, 52816–52819 (2004).

4. Mokhonov, V., et al. Multidrug transporter MexB of Pseudomonas aeruginosa: overexpression, purification, and initial structural characterization. *Protein Expr. Purif.* **40**, 91–100 (2005).
