# Supplementary material for: Structures of the wild-type MexAB–OprM tripartite pump reveal its complex formation and drug efflux mechanism
Source: Nat Commun. 2019 Apr 3;10:1520. doi: 10.1038/s41467-019-09463-9 (PMC6447562; doi:10.1038/s41467-019-09463-9)
Supplement: Supplementary file 5 — Description of Additional Supplementary Files [file 41467_2019_9463_MOESM5_ESM.docx]

**Title:** Supplementary Movie 1
**Description:** Side view of a model of OprM channel opening.

**Title:** Supplementary Movie 2
**Description:** Top view of a model of OprM channel opening.
